# hESC-secreted proteins can be enriched for multiple regenerative therapies by heparin-binding

**DOI:** 10.18632/aging.100559

**Published:** 2013-05-23

**Authors:** Hanadie Yousef, Michael J. Conboy, Ju Li, Matthew Zeiderman, Tandis Vazin, Christina Schlesinger, David V. Schaffer, Irina M. Conboy

**Affiliations:** ^1^ Department of Bioengineering and California Institute for Quantitative Biosciences (QB3), UC Berkeley, Berkeley, CA 94720, USA; ^2^ Department of Molecular and Cellular Biology, UC Berkeley, Berkeley, CA 94720, USA; ^3^ Department of Chemical Engineering and Helen Wills Neuroscience Institute, UC Berkeley, Berkeley, CA 94720 USA

**Keywords:** rejuvenation, embryonic stem cell, myoblast, satellite cell

## Abstract

This work builds upon our findings that proteins secreted by hESCs exhibit pro-regenerative activity, and determines that hESC-conditioned medium robustly enhances the proliferation of both muscle and neural progenitor cells. Importantly, this work establishes that it is the proteins that bind heparin which are responsible for the pro-myogenic effects of hESC-conditioned medium, and indicates that this strategy is suitable for enriching the potentially therapeutic factors. Additionally, this work shows that hESC-secreted proteins act independently of the mitogen FGF-2, and suggests that FGF-2 is unlikely to be a pro-aging molecule in the physiological decline of old muscle repair. Moreover, hESC-secreted factors improve the viability of human cortical neurons in an Alzheimer's disease (AD) model, suggesting that these factors can enhance the maintenance and regeneration of multiple tissues in the aging body.

## INTRODUCTION

Tissue regeneration and maintenance dramatically and invariably decline with age, eventually causing failure of multiple organ systems in all mammals. In muscle, the loss of tissue regeneration with age is thought to be imposed by signaling changes in the satellite stem cell niche, and interestingly, the aging of stem cell niches is to some extent similar between muscle, brain, blood, and other tissues [1-3]. Our previous work found that human embryonic stem cells (hESCs) produce soluble secreted molecules that can counteract the age-imposed inhibition of muscle regeneration, an “anti-aging” activity that is lost when the hESCs differentiate [4, 5].

Numerous mitogenic proteins are expressed by hESCs [6] and are known to act through TGF-beta/BMP, Jak-Stat, MAPK, and other key regulatory signaling pathways, all of which have been implicated in the control of adult tissue regeneration. The precise identity of the pro-myogenic factors that are secreted by hESCs and the molecular mechanism of their action in muscle stem and progenitor cells is still work in progress; however, the effects of one of these molecules, FGF-2, was studied here in detail. FGF-2 is known to be secreted by hESCs and is also contained in the growth/expansion medium of embryonic stem cells [7-9]. FGF-2 does not have a signal peptide and is not secreted through the ER-Golgi pathway [10, 11], and the mechanisms of FGF-2 transport or export from cells in skeletal muscle are not well defined. FGF-2 ligand acts by binding to promiscuous receptor complexes to activate the MAPK/PERK pathway, which is well known to exert strong mitogenic effects and to be necessary for the establishment and maintenance of primary cultures of muscle progenitor cells [12, 13].

With age, the activation and proliferation response of aged muscle stem cells after injury declines as compared to young [14]. Consequentially, the generation of fusion-competent muscle progenitor cells, or myoblasts, that co-express desmin, Myf5, MyoD and Pax-7, incorporate BrdU, and terminally differentiate into myotubes or myofibers that express eMyHC, becomes deficient in poorly regenerating old tissue [1]. Controversially, a recent report [15] suggested that FGF-2 is overproduced by aged myofibers and subsequently induces proliferation and exhaustion of the old satellite cells that are typically quiescent. The age-specific role of FGF-2 was examined here with respect to its localization and signaling in muscle stem cells.

The age-imposed decline in stem cell responses is caused by the aging of the niche, not only in muscle, but also in brain. Thus, we tested whether the enhancement of stem and progenitor proliferation and tissue maintenance by hESC-secreted proteins is conserved between muscle and brain. The brain undergoes many changes with aging, including neuronal cell death, thinning of the cortex and loss of brain plasticity, and the accumulation of plaques and neurofibrillary tangles [16-19]. Additionally, two regions of the adult brain - the dentate gyrus of the hippocampus and the subventricular zone of the forebrain - harbor neural stem cells (NSCs) that express the marker Sox-2 and are able to give rise to neurons and glia in vivo and in culture. During the natural aging process, similarly to muscle function, cognitive function declines, which may be in part due to the diminished ability of the neural stem cells in the subgranular zone of the dentate gyrus to proliferate and give rise to new neurons [2, 3].

In addition to decrease in neurogenesis observed with aging, the central nervous system can suffer from a number of age-associated neurodegenerative disorders. We have developed an *in vitro* Alzheimer's disease model in which hESC-derived cortical neurons are exposed to a very toxic form of Amyloid beta (Aβ) (Vazin et al., submitted), soluble oligomeric forms of Aβ known as “globulomers”, which have shown a stronger clinical correlation with the cognitive deficit than the overall plaque load [20, 21]. Exposure of hESC-derived glutamatergic neurons to such Aβ oligomers induces signs of the disease, including age-dependent binding of Aβ and cell death.

In investigating the pro-myogenic properties of hESC-secreted proteins, we explored a hypothesis that key factors may contain heparin-binding domains, as many proteins known to be key mitogenic regulators of cell-fate specification and secreted by embryonic cells bind heparin or act in complex with heparin-bound proteins [22, 23]. Consistent with this hypothesis, we establish that that depletion of the heparin-binding proteins abrogates, while the enrichment for these proteins robustly manifests, the pro-regenerative activity of the hESC-conditioned medium. In addition to providing a novel method for enrichment of the therapeutic factors that are secreted by the hESCs, this study demonstrates the positive effect of these molecules on tissue regeneration and maintenance not only in muscle, but also in brain. Namely, hESC-secreted proteins robustly enhanced the proliferation of adult NSCs, suggesting a promising application for both the enhancement of cognitive function and improved outcome of NPCs transplantation; and notably, proteins secreted by hESCs had significant neuroprotective, anti-apoptotic effect on human cortical neurons exposed to ab, demonstrating a potential novel therapy for combating AD. Importantly, f this work establishes that hESC-secreted proteins act independently of recombinant FGF-2 that is contained in their growth medium. Interestingly, we also show that mTeSR-1 hESC-conditioned medium exhibits potent pro-myogenic properties due to the high levels of FGF-2. In FGF-2 is not a pro-aging molecule, our work demonstrates that FGF-2 does not signal in the aged muscle stem cells and uncovers an interesting, age-specific mis-localization of the FGF-2 ligand, which may reflect a fundamental difference not only in the permissiveness of FGF-2 signaling in young vs. old muscle, but also in the ability of old differentiated muscle cells to secrete this mitogen.

## RESULTS AND DISCUSSION

### mTeSR-1 growth medium has pro-myogenic activity, which is due to the high levels of FGF-2, and hESC-secreted factors act independently of recombinant FGF-2

**Figure 1:**
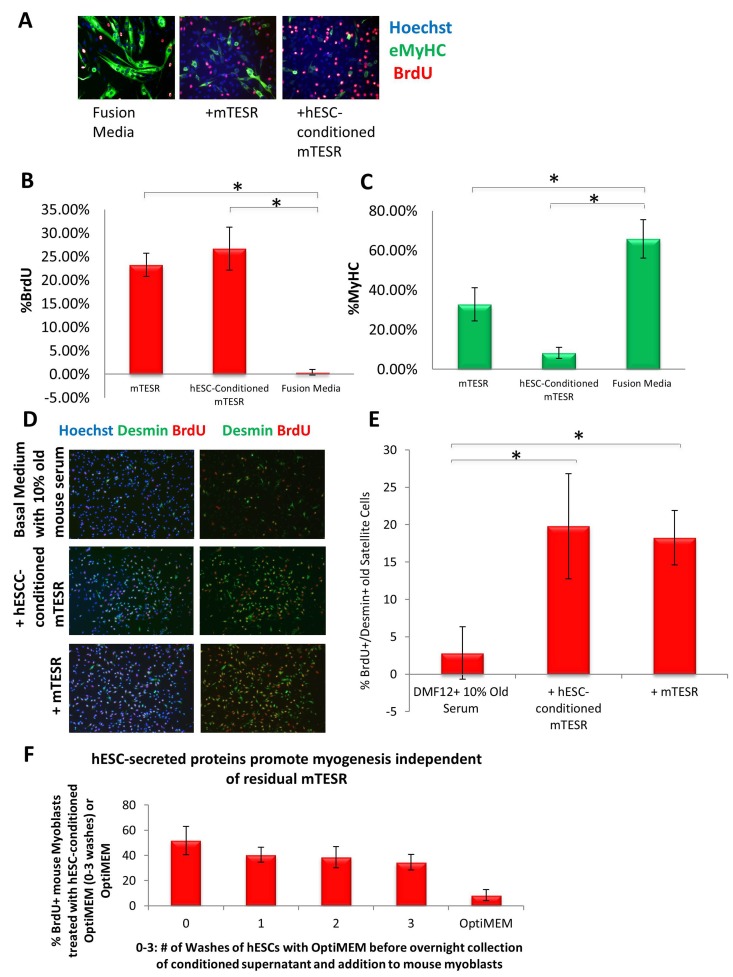
Both mTeSR-1 and hESC-Conditioned mTeSR-1 increase primary myoblast and satellite cell Proliferation and inhibit Differentiation (**A**) Primary Mouse Myoblasts were cultured for 24 hours in 50% fusion/differentiation medium (DMEM, 2% horse serum) plus 50% of the specified medium. A 2 hour BrdU pulse was performed before cell fixation to label proliferating cells. Immunofluorescence was performed for eMyHC (green) and BrdU (red), with Hoechst (blue) labeling all nuclei. Representative images are shown. Proliferation and differentiation of fusion-competent myoblasts were quantified by cell scoring in 25 random fields of each condition using a Molecular Devices automated imager and MetaXpress cell scoring software. Results are displayed as the mean percent of BrdU+ (**B**) or eMyHC+ (**C**) proliferating or differentiating cells +/−SD, respectively. N=4 *P< 4×10^−10^ for BrdU+ myoblasts -incubated in 50% mTeSR-1 as compared to myoblasts incubated in just fusion medium, or 50% hESC-conditioned mTeSR-1 as compared to myoblasts incubated in just fusion medium. *P< 0.005 for eMyHC+ fusing myoblasts in 50% mTeSR-1 as compared to myo-blasts incubated in fusion medium alone, and *P< 9×10-5 for eMyHC+ fusing myoblasts in 50% hESC-conditioned mTeSR-1 as compared to myoblasts incubated in just fusion medium. (D) Old injury activated myofiber-associated satellite cells were isolated at 3 days post cardiotoxin-induced muscle injury, and cultured overnight in 50% DMEM/F12 with 10% old serum, and 50% of the medium specified, followed by a 2 hour BrdU pulse to label proliferating cells before cell fixation. Immunofluorescence was performed with Desmin (green) and BrdU (red), with Hoechst (blue) labeling all cell nuclei. Representative images are shown and demonstrate that both hESCconditioned mTeSR-1 and mTeSR-1 have a pro-myogenic effect on activated satellite cells. (E) Proliferating Desmin+/BrdU+ satellite cells were quantified by cell scoring in multiple random fields of each condition. Results are displayed as the mean percent of BrdU+/Desmin+ proliferating satellite cell cells +/−SD. N=3, *P< 0.05 for satellite cells in 50% mTeSR-1 as compared to satellite cells incubated in just basal medium with old serum, and *P< 0.001 for satellite cells in 50% hESC-conditioned mTeSR-1 as compared to satellite cells incubated in just basal medium with old serum. (F) Undifferentiated hESCs that were grown in mTeSR-1 medium were washed 0-3 times with Opti-MEM, followed by overnight incubation in Opti-MEM and collection of the resulting conditioned Opti-MEM. The hESC-conditioned Opti-MEM was spun down to remove cell debris, before addition to myoblasts as a 50/50 mix with myogenic fusion medium for culture overnight. A 2 hour BrdU pulse was performed to label proliferating cells prior to cell fixation and immunofluorescence was performed with eMyHC and BrdU, with Hoechst labeling all cell nuclei (images not shown). Proliferating and differentiating cells were quantified by cell scoring 25 random fields of each condition using an automated imager and MetaXpress cell scoring software. Results are displayed as the mean percent of BrdU+ or eMyHC+ proliferating or differentiating cells +/−SD, respectively. N=2

Our previous work established that injection of hESCs - which were cultured on mouse embryonic fibroblasts (MEF) and in standard, highly mitogenic, embryonic cell growth medium - enhanced old muscle regeneration [4]. In our more recent work, the hESCs have been cultured in mTeSR-1 (Stem Cell Technologies), a defined feeder-free medium which is also highly mitogenic [9], and we investigated whether and to what degree the pro-myogenic effects of hESC-conditioned medium was due to the residual activity of the hESC growth/expansion medium. Primary muscle progenitor cells (myoblasts) were cultured overnight in a mitogen-low fusion medium that typically induces differentiation of myoblasts into multinucleated eMyHC+ myotubes. The enhancement of myogenic cell proliferation and inhibition of differentiation was assayed by BrdU uptake for the last 2 hours of culture, after which cells were fixed and used for immuno-fluorescence with anti-BrdU and anti-MyHC specific antibodies. When primary myoblasts were cultured in 50% fusion medium plus 50% hESC-conditioned mTeSR-1 or 50% unconditioned mTeSR-1, both media compositions induced proliferation and inhibited differentiation of these myogenic cells, though medium containing hESC-conditioned mTeSR-1 inhibited differentiation more significantly (Figure 1A, quantified in B and C). To confirm these data with muscle stem cells, injury-activated satellite cells associated with myofibers were isolated from old muscle and cultured overnight in a 50/50 mix of Opti-MEM containing 5% old mouse serum and hESC-conditioned mTeSR-1 or mTeSR-1. Both conditioned and not-conditioned mTeSR-1 media enhanced the regenerative capacity of satellite cells that were isolated from injured old muscle, based on the numbers of de-novo generated BrdU+/Desmin+ muscle progenitor cells (Figure 1D, quantified in E). These results demonstrate that embryonic stem cell culture medium itself has pro-myogenic effects. To investigate whether hESC-conditioned Opti-MEM exhibits pro-regenerative effects due to the hESC-secreted proteins, and not because of residual mTeSR-1, we washed the hESC culture wells multiple times with Opti-MEM prior to incubation for conditioning the Opti-MEM, and found that even after 3 washes, hESC conditioned the Opti-MEM to yield the same potent pro-regenerative effect on myoblasts (Figure 1F). These results demonstrate that while mTeSR-1 supplementation promotes myoblast proliferation, other factor(s) produced by hESCs independently enhance the regenerative capacity of muscle stem and progenitor cells.

**Figure 2:**
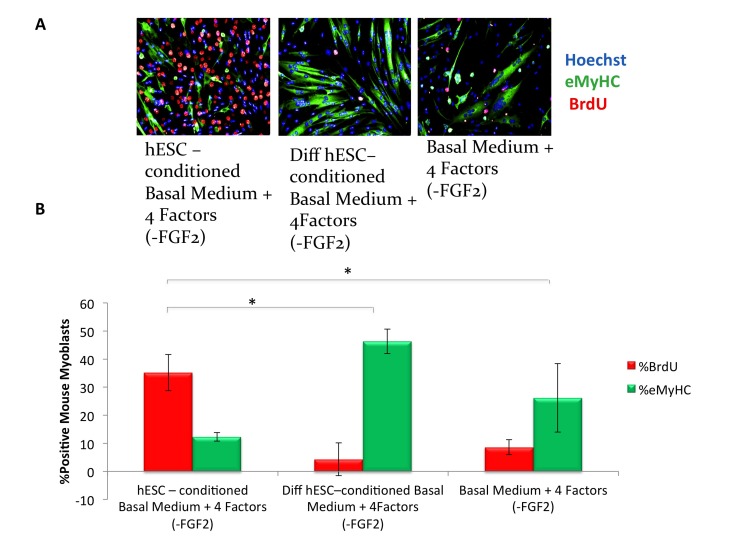
hESC-conditioned medium enhances myogenic proliferation in the absence of FGF2 in mTeSR-1 growth medium (**A**) Primary myoblasts were cultured for 16 hours in 50% fusion/differentiation Medium + 50% of the specified medium. A 2 hour BrdU pulse was performed before cell fixation to label proliferating cells. Immunofluorescence was performed for eMyHC (green) and BrdU (red), with Hoechst (blue) labeling all nuclei. Representative images demonstrate that hESC-conditioned medium lacking FGF2 increases myoblast proliferation and inhibits differentiation. (**B**) Proliferation and differentiation of fusion-competent myoblasts were quantified by cell scoring in 50 random fields of each condition using an automated imager and MetaXpress cell scoring software. Results are displayed as the mean percent of BrdU+ or eMyHC+ proliferating or differentiating cells +/−SD, respectively. N=4, *P< 2×10^−12^ for hESC-conditioned basal medium with 4 mTeSR-1 ingredient components (lacking FGF2) as compared to differentiation hESC-conditioned basal medium with 4 mTeSR-1 ingredient components (also lacking FGF2), and for hESC-conditioned basal medium with 4 mTeSR-1 ingredient components (lacking FGF2) as compared to myoblasts incubated in basal medium with 4 mTeSR-1 ingredient components (lacking FGF2).

To understand the pro-myogenic effects of mTeSR-1 in greater detail, we addressed the role of FGF-2, which is present at high concentration in mTeSR-1 (over 50 nanograms per milliliter, ~10 times higher than the doses used in conventional culture of muscle progenitor cells). Our hypothesis was that the FGF-2 in mTeSR-1 enhances myoblast and satellite cell proliferation, partially masking the effects of the hESC-produced factors in hESC-conditioned mTeSR-1. To test this hypothesis, we incubated hESCs in a basal medium that had the other growth and signaling factors present in mTeSR-1 (TGF-beta, GABA, pipecolic acid and Lithium Chloride, [9]), but lacked FGF2, and compared the pro-myogenic effects of this FGF-free hESC-conditioned mTeSR-1 analog with the effects of the same mTeSR-1 analog that was not conditioned by the hESCs. Without FGF-2, the mTeSR-1 analog lacked pro-regenerative effects on myoblasts (Figure 2A, quantified in 2B). On the other hand the very same mTeSR-1 analog lacking FGF-2, but conditioned by hESCs, significantly enhanced myoblast proliferation and inhibited differentiation, while conditioning of this mTeSR-1 analog lacking FGF-2 by differentiated hESC derived cells resulted in the absence of pro-myogenic properties (Figure 2A, quantified in 2B). These data demonstrate that the pro-myogenic effects of mTeSR-1 are due to the high concentration of FGF-2, and that it is not simply residual FGF-2 from mTeSR-1 that is responsible for the enhancement of myogenesis by the hESC-conditioned medium.

### FGF-2 signaling and satellite cell proliferation are not increased with age

**Figure 3:**
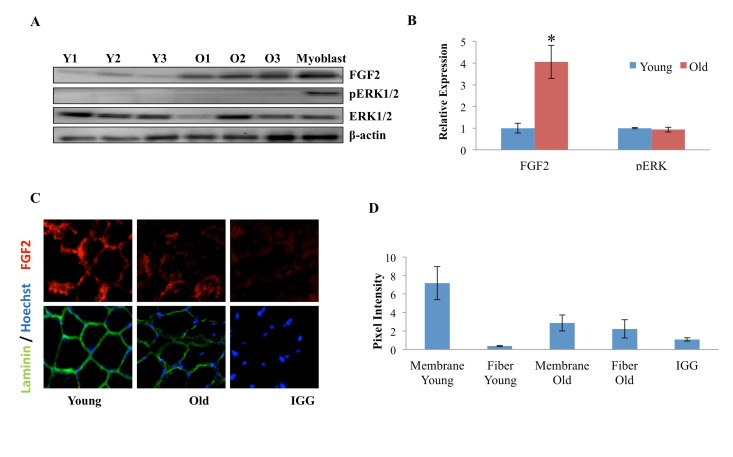
Age-dependent comparison of FGF2 and pERK levels and localization in muscle fibers (**A**) Protein was isolated from freshly-derived uninjured myofibers of young and old mice and the levels of FGF2 and phospho-ERK1/2; total ERK1/2 and cytoplasmic beta-actin were analyzed by Western blotting, using specific antibodies. Representative data are shown. (**B**) Relative protein expression was quantified in 3 young and 3 old mice by normalization of FGF-2 to beta-actin and normalization of pERK to total ERK; significantly higher levels of FGF-2, but not of pERK were detected in the old myofibers, as compared to young (n=3, * P<0.05). (**C**) Tibialis anterior (TA) muscle from 2 young and 2 old mice were sectioned and immunostained for laminin (green) and FGF2 (red). Hoechst (blue) labels all nuclei. Representative images demonstrate the presence of FGF-2 and laminin in muscle compartments, as compared to the negative IgG control and higher FGF-2 levels seem to be present in the laminin+ basement membranes of the young myofibers, as compared to old. (**D**). The pixel density of FGF-2 that co-localizes with laminin+ basement membrane vs. the internal regions of the myofibers was determined in 30-40 areas of each cryosection of 3 muscle tissue slides from young and old muscle, using Image J software. Preferential localization of FGF-2 in the basement membrane was identified in young muscle, while in the old tissue, FGF-2 was mis-localized to the center of the myofibers and away from the basement membrane, n=3, * P<0.05.

FGF-2, which often functions as a mitogen, was recently reported to contribute to the aging and depletion of mouse satellite cells. However, the canonical model of muscle stem cell aging postulates that a decline in such mitogens over time leads to reduced activation of satellite cells that are resident to old tissue [1, 24, 25], so we explored these phenomena in more detail. The levels of FGF-2 were determined by Western Blotting in muscle fibers that were derived from Tibialis Anterior (TA) and Gastrocnemius (Gastroc) muscle of young and old mice. As shown in Figure 3A (quantified in 3B), a significant increase in FGF-2 protein was observed with age in myofibers, consistent with Chakkalakal et al. FGF-2 signals through the MAPK/pERK pathway, so we analyzed the levels of pERK in myofibers derived from young and old uninjured muscle. Interestingly, as shown in Figure 3 A (quantified in 3B), no age-specific increase in pERK was found, and the levels of this key effector were very low in cells from both ages, despite the high levels of FGF-2 in protein lysates derived from old muscle fibers. Also, a myoblast control indicates that pERK detection was sensitive (Figure 3A). To under-stand these data, we examined the presence and localization of FGF-2 in the intact young and old muscle, using 10 micron cryosections. FGF-2 and laminin were detected with specific antibodies and resolved by immunofluorescence. As shown in Figure 3 C (quantified in 3D), FGF-2 was localized in the basement membrane of young muscle, while in the old muscle, FGF-2 was present less in the basement membrane and more in the cytoplasm of the myofibers (e.g. away from its receptors in muscle stem cells). These data suggest that the relatively higher levels of FGF-2 in old muscle do not necessarily represent ligand that is available for signaling in satellite cells. Additionally, these results indicate that detection of elevated FGF-2 in the old muscle might be due to its over-expression within the old muscle fiber itself, or alternatively, due to “washing” of extracellular FGF-2 from young muscle during tissue dissociation when the basement membrane is digested with collagenase and dispase, and tissue integrity is perturbed [26].

**Figure 4:**
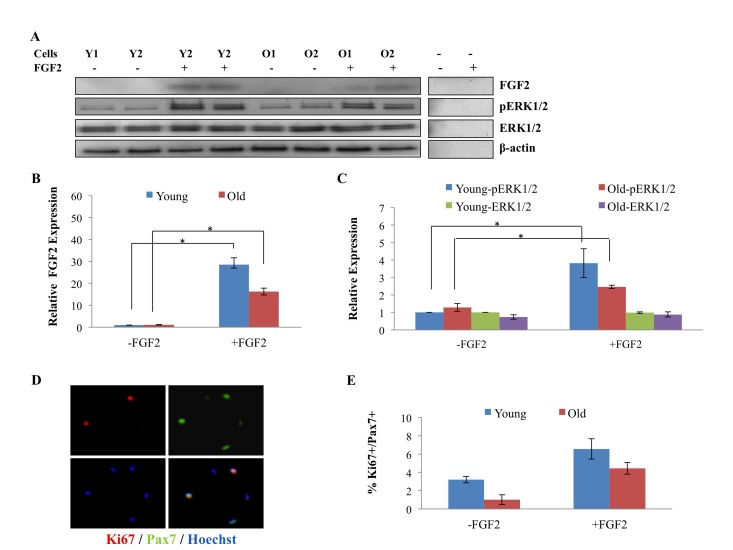
Age-related comparison of FGF2 and pERK levels in muscle stem cells derived from uninjured tissue and of proliferation of these cells (**A**) Quiescent muscle stem cells were isolated from uninjured young and old muscle as described in Methods. The cells were treated (or not) with FGF2 (10ng/ml) for 30 minutes before being lysed and analyzed for the levels of FGF2, phospho-ERK1/2, total ERK1/2 and beta actin by Western Blotting. Representative images are shown. (**B**) Relative protein expression of FGF-2, pERK and total ERK were quantified from 3 young and 3 old mice, using beta-actin for normalization. The levels of FGF-2 were equally undetectable in young and old satellite cells, however, added FGF-2 was clearly detected in the cells of both ages after ~2min exposure (but was not detected in accelular samples even after 10min exposure); the levels of pERK and total were equally low in young and old satellite cells and pERK, but not total ERK was, as expected, induced by added FGF-2. n=3, * P<0.05. (**C**) Muscle stem cells from resting muscle were treated (or not) with FGF2 (10ng/ml) for 24 hours before immunostaining for Ki67 and Pax7. Percent of Ki67+/Pax7+ proliferating myogenic cells were quantified. No age-specific increase in cell proliferation was detected in satellite cells isolated from old uninjured muscle, and in contrast, more proliferating satellite cells were observed in the cultures derived from young muscle. Added FGF-2 enhanced the proliferation of both young and old muscle stem cells in these overnight cultures. n=3, * P<0.05.

To confirm and extend upon these findings, we isolated muscle stem cells from uninjured young and old TA and Gastroc muscle and treated them with FGF-2 for 30 minutes, after which the levels of FGF-2, pERK, and total ERK were determined in these freshly isolated stem cells. As shown in Figure 4 A, B, endogenous FGF-2 was undetectable in either young or old muscle stem cells upon isolation, but the added FGF-2 was clearly present in these satellite cells after 30 minutes. Young and old satellite cells were harvested after just 30 minutes of culture, thus, the FGF-2 protein detected in cultures, which were treated with recombinant FGF-2 is unlikely to represent de-novo expression. Satellite cells were lifted from the plates with PBS and washed prior to their lysing for Western Blotting, and it was thus unlikely that any residual, non-cell associated recombinant FGF-2 from media or plates would contaminate cell lysates. To test this directly and definitively, we performed a control with a matrix-coated but cell-free plate that was identically treated with FGF-2, and found no detectable recombinant FGF-2 in the solution (Figure 4A). Hence, the FGF-2 detected in protein lysates of young and old satellite cells incubated with this growth factor likely reflects ligand that is bound to its specific receptors. In support of this conclusion, recombinant FGF-2 induced pERK in both young and old satellite cells (Figure 4 A and C). In agreement with non-detectable endogenous FGF-2 in both young and old satellite cells, very low levels of pERK that did not differ with age were observed in these muscle stem cells resident to tissue that was neither injured nor treated with recombinant FGF-2 (Figure 4 A and C). To determine whether low levels (as opposed to none) of FGF-2 can be detected in the muscle stem cells, another independent experiment was performed with a prolonged enhanced chemiluminescence exposure of the Western Blots. As shown in Supplementary Figure 1, low levels of FGF-2 could be indeed detected in muscle stem cells after a 30 minute exposure, but once again, there was no age-specific difference in either FGF-2 or in pERK. These results suggest that FGF-2 does not signal in either young or old satellite cells that reside in non-injured skeletal muscle.

To directly examine cell proliferation, satellite cells were isolated from non-injured young and old tissue and were cultured with or without FGF-2 overnight, after which the levels of the proliferation marker Ki67 were determined in Pax7+ satellite cells. Muscle stem cells for this and other experiments were isolated with high and equal purity from young and old mice, as shown in Supplementary Figure 2. Neither young nor old cells were lost during overnight culturing, as the numbers were similar to initial plating, and no age-specific loss was observed, based on the cell counts. As shown in Figure 4 D and E, no increase in proliferation of aged muscle stem cells was detected, as compared to young, and as expected from previous literature, the majority of both young and old satellite cells were quiescent [14, 26, 27]. When added, FGF-2 significantly enhanced the proliferation of quiescent muscle stem cells that were isolated from uninjured muscle (both young and old), as shown in Figure 4 D and E, which is consistent with the induction of pERK that is shown in Figure 4 A, C. However, very interestingly, 90-95% of muscle stem cells derived from uninjured young and old tissue were not proliferating even in the presence of added FGF-2, suggesting that other mitogens and / or cell-fate changes are needed to induce the robust entry of quiescent satellite cells into the cell cycle, also as published [13]. These data demonstrate that the localization of FGF-2 within the skeletal muscle compartment changes with age and question whether endogenous FGF-2 is likely to exhaust the pool of aged quiescent satellite cells, since it does not induce significant signaling in these cells.

### The pro-regenerative activity of hESC-secreted factors is contained in proteins with heparin binding domains

Based on the fact that many growth factors that are known to enhance cell proliferation contain heparin binding domains, or act by association with heparin binding proteins as co-activators of signal transduction [22, 23], we hypothesized that hESC-secreted factors that have pro-regenerative activity may be proteins that might bind heparin, and furthermore postulated that hESC-conditioned medium depleted of heparin-binding proteins would lose the ability to enhance myoblast proliferation. To confirm that the factors in hESC conditioned medium were proteins, hESC conditioned Opti-MEM was treated with proteinase-K agarose beads, and the beads were removed before mixing 50/50 with Opti-MEM and 5% mouse serum, for culture with injury-activated satellite cells with associated fibers from old muscle, as above. All proliferative activity of the conditioned medium was lost after proteinase treatment, indicating that protein(s) conferred the pro-regenerative activity (Supplemental Figure 3).

**Figure 5:**
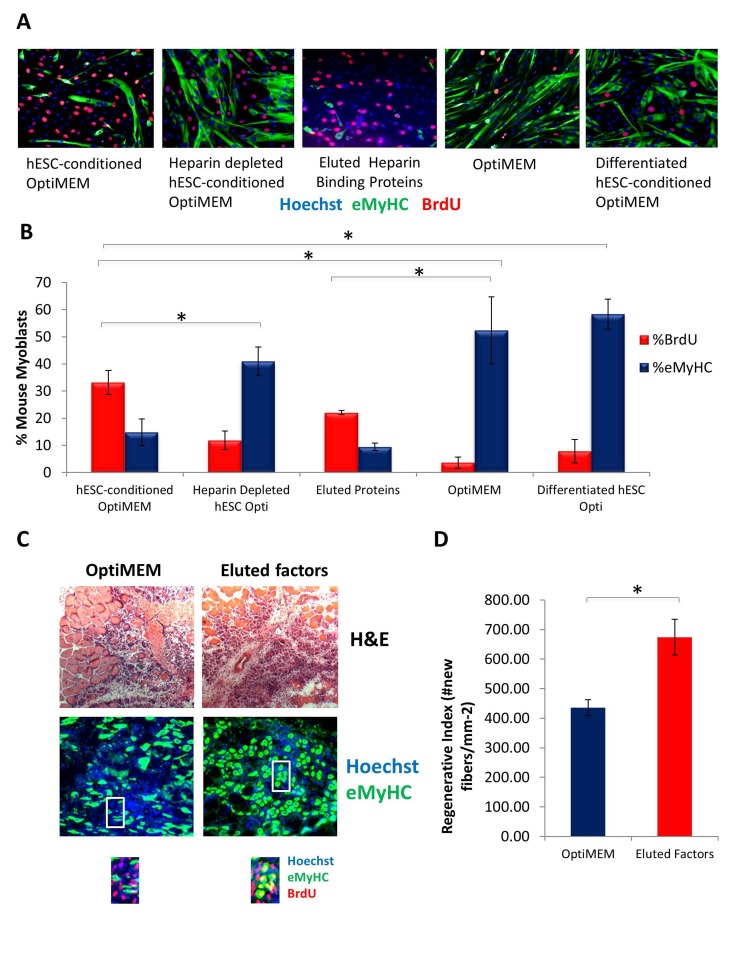
Pro-regenerative Embryonic Factors Contain Heparin Binding Domains (**A**) Primary Mouse Myoblasts were cultured for 24 hours in 50% fusion/differentiation medium + 50% of the specified medium. A 2 hour BrdU pulse was performed before cell fixation to label proliferating cells. Immuno-fluorescence was performed for eMyHC (green) and BrdU (red), with Hoechst (blue) labeling all nuclei. Representative images are shown. (**B**) Proliferation and differentiation of fusion-competent myoblasts were quantified by cell scoring in 25-100 random fields of each condition using an automated imager and MetaXpress cell scoring software. Results are displayed as the mean percent of BrdU+ or eMyHC+ proliferating or differentiating cells +/−SD, respectively. N=6 *P< 3×10^^−45^ for hESC-conditioned Opti-MEM compared to differentiated hESC-conditioned Opti-MEM, and hESC-conditioned Opti-MEM compared to Opti-MEM. *P<0.005 for hESC-conditioned Opti-MEM compared to heparin depleted hESC-conditioned Opti-MEM, and hESC-conditioned Opti-MEM compared to differentiated hESC-conditioned OptiMEM. *P< 5×10^^−7^ for hESC-conditioned Opti-MEM compared to Opti-MEM, and Eluted Proteins compared to Opti-MEM. (**C**) Old Tibialis Anterior muscles were injured with cardiotoxin (see Methods). Heparin bound and eluted protein or vehicle control (Opti-MEM) were injected into sites of injury on Day 0 and Day 2. BrdU was injected (intraperitoneal) at 3 days post injury to label proliferating, fusion-competent myoblasts. Animals were sacrificed and muscle was collected 5 days post injury. Cryosections (10 ?m) were analyzed by hematoxylin/eosin (H&E) staining and immunostaining for embryonic myosin heavy chain (eMyHC, shown in green) and BrdU incorporation (shown in red). Hoechst stains nuclei (blue). As shown by representative images, the regenerative outcome of old muscle given eluted factors was significantly improved as compared to old muscle given Opti-MEM vehicle control, based on significantly diminished scar tissue formation, larger and more dense *de novo* myofibers and an increase in the numbers of eMyHC+ myofibers with centrally-located BrdU+ nuclei that replaced the damaged tissue. (**D**) Regeneration of old mouse Tibialis Anterior 5 days post injury, that received eluted factors or vehicle, was quantified from muscle sections, and is presented as the number of newly regenerated myofibers per square millimeter of injury site. Error bars indicate SD, n=3 mice per group. *P <0.02 between old given eluted factors and old given vehicle control.

To deplete heparin-binding proteins, hESC-conditioned medium was incubated with heparin binding domain - coated acrylic beads. Muscle progenitor cells were then cultured in this heparin-depleted hESC-conditioned medium, hESC-conditioned medium, or controls (medium alone and medium conditioned by differentiated cells that lack the pro-regenerative activity). Proliferation of primary muscle progenitor cells was assayed by BrdU uptake for 2 hours, and cell differentiation was assayed by the expression of eMyHC. Interestingly, hESC-conditioned medium depleted of heparin binding proteins completely lost its pro-regenerative activity on muscle progenitor cells (Figure 5 A, quantified in B). Even more importantly, the pro-regenerative activity of in the hESC-secreted proteins could be eluted from the heparin-coated beads (Figure 5 A, quantified in B), hence confirming that these factors have heparin-binding domains and suggesting novel strategies for purification of these clinically relevant molecules. Excitingly, when these heparin-binding eluted embryonic proteins were injected at Day 0 and Day 2 into injured muscle (e.g., at the time of the injury and when muscle stem cells become activated for regeneration) old muscle repair became rejuvenated, based on increased formation of de-novo myofibers with centrally located BrdU+ nuclei (Figure 5C, quantified in D). These data reveal the pro-myogenic proteins that are secreted by the hESCs contain heparin-binding domains.

### hESC-conditioned Opti-MEM has Pro-Survival and Pro-Mitogenic effects on Neuronal Cell Types

**Figure 6:**
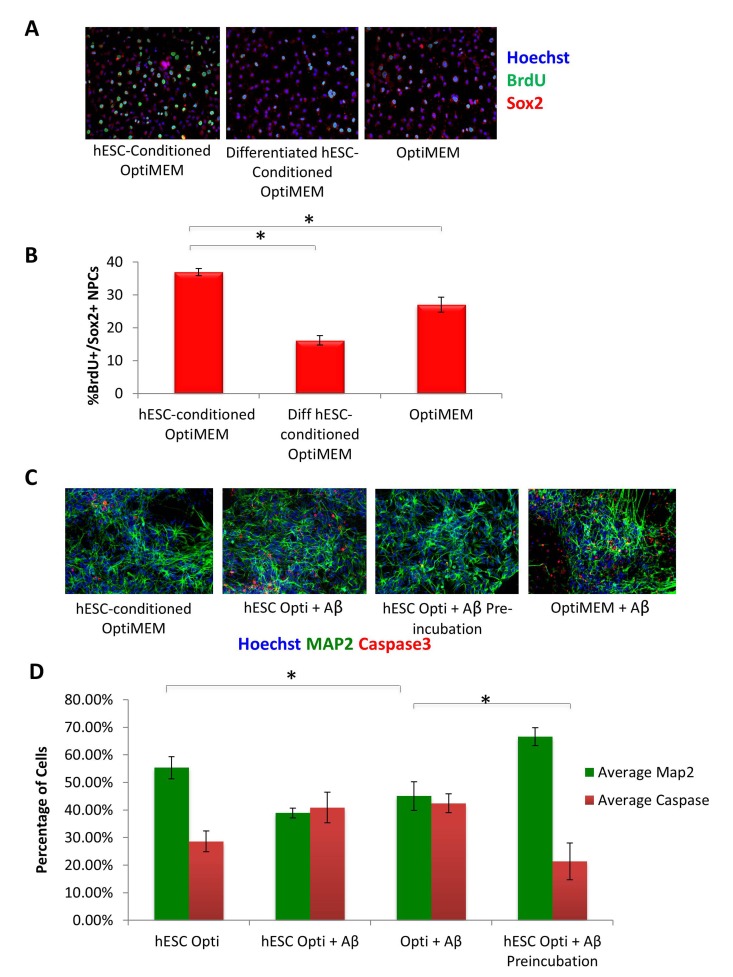
hESC-secreted Factors Enhance NPC Proliferation and are Neuroprotective (**A**) Rat Neural Progenitor Cells (rNPCs) were cultured overnight in 50% differentiation medium (DMF12 + N2) and 50% specified medium followed by a 4 hour BrdU pulse to label proliferating cells prior to fixation. Immunofluorescence was performed for Sox2 (red) and BrdU (green), with Hoechst (blue) labeling cell nuclei. Representative images are shown. (**B**) Quantification of BrdU+/Sox2+ proliferating cells was performed by cell scoring in 100 random fields of each condition using an automated imager and MetaXpress cell scoring software. Results are displayed as the mean percent of BrdU+/Sox2+ proliferating cells +/−SD; N=4, *P< 2×10^−15^ for rNPCs incubated in hESC-conditioned Opti-MEM as compared to rNPCs incubated in differentiated hESC-conditioned Opti-MEM, and *P< 0.0002 for rNPCs incubated in hESC-conditioned Opti-MEM as compared to rNPCs incubated in Opti-MEM. (**C**) Pre-incubation of Aβ globulomers with hESC -conditioned Opti-MEM before incubation with mature cortical neurons prevents neuron cell death and exhibits a neurotrophic effect, as shown via decreased immunofluorescence staining of cleaved caspase3 (red) and increased Map2 + (green) neurons. Hoechst (blue) labels all nuclei. Representative images are shown. (**D**) Total number of Map2+ neurons and the amount of apoptosis was quantified by cell scoring of random fields taken by an automated imager of each condition in the above assay performed in replicates. Results are displayed as the mean percent of caspase + or Map2+ (**C**) proliferating or differentiating cells +/−SD, respectively. N=4, *P< 0.02 for Map2+ cortical neurons treated with Aβ globulomers preincubated in hESC-conditioned Opti-MEM, as compared to treatment with Aβ globulomers in OptiMEM, and *P< 0.05 for the level of caspase3 in cortical neurons treated with Aβ globulomers preincubated in hESC-conditioned Opti-MEM, as compared to treatment with Aβ globulomers in Opti-MEM.

To assess the potential positive effect of hESC-secreted proteins on other cell types, specifically neural cells, we cultured rat neural progenitor cells in the presence of hESC-conditioned medium, or in a control medium conditioned by differentiated hESC-derived cells. Specifically, cells were cultured in the 50/50 mix of neural differentiation medium (see Methods) and Opti-MEM, which was conditioned either by the self-renewing hESCs or by the negative control, differentiated hESC-derived cells. The goal was to determine if hESC-secreted factors can enhance proliferation and inhibit differentiation of NPCs, in parallel to our studies demonstrating these embryonic factors enhance muscle precursor proliferation and inhibit their differentiation in a 50/50 mix of fusion medium [5]. Very interestingly, a significant increase in proliferation of Sox-2+ neural progenitors was observed in cultures exposed to the hESC-produced proteins, an effect that was lost when NPCs were cultured in control medium from differentiated cells (Figure 6 A, quantified in B). As this effect was similar to what we previously reported for muscle stem/progenitor cells, in that we observe an enhancement of proliferation and inhibition of differentiation of precursor cells by hESC-secreted factors [5], it suggests that hESC-secreted proteins enhance the proliferative capacity of progenitor cells in multiple tissue types, and similarly to the situation in muscle, the pro-mitogenic activity is lost when hESCs differentiate.

We next sought to examine whether not only cell proliferation, but cell viability might be enhanced by the hESC-secreted proteins, particularly under pathological conditions. Likewise, we wished to investigate whether the effects of the pro-mitogenic factors would manifest not only on progenitors, but also on terminally differentiated neurons. To answer these questions, we generated human cortical glutamatergic neurons by directed differentiation of embryonic stem cells (see Methods). Specifically, dorsal telencephalic progenitors expressing glutamate and VgluT1 were generated by using Shh and FGF-2. This protocol induced the differentiation of human embryonic stem cells (hESCs) into cultures with up to 74% of neurons expressing glutamate and VgluT1. As an *in vitro* model of AD, soluble oligomeric forms of Aβ known as “globule-mers,” which have been implicated in the pathology of Alzheimer's disease [20, 21], were added to these cultures of human glutamatergic neurons. They bound Aβ, which led to cell death as measured by the presence of cleaved caspase 3 (Figure 6C, quantified in D).

To examine whether hESC-secreted factors have neuroprotective effects in this *in vitro* human AD model, Aβ globulomers were added to cortical cultures primarily comprised of glutamatergic neurons in the presence or absence of hESC-conditioned medium. The neurons were pre-incubated with hESC-conditioned Opti-MEM for 1 hr prior to treatment with Aβ globulomers, or alternatively, hESC-conditioned medium was added at 50% to neuron medium, simultaneously with the Aβ globulomers. Analysis with cleaved caspase-3 as an apoptotic marker and MAP2 as neuron marker showed a significant decrease in cell death when neurons were pre-incubated with hESC-secreted factors, as compared to cultures treated with Aβ globulomers alone (Figure 6 C, quantified in D). A noticeable but not statistically significant decrease of apoptosis was observed in neuron cultures that were administered with Aβ and hESC-secreted proteins simultaneously (Figure 6 C, D). These data suggest that hESC-secreted factors exert a protective (anti-apoptotic) effect on human cortical neurons in this AD model.

### Conclusions

Since the comprehensive molecular identity of the specific proteins that are responsible for the pro-regenerative effects of the hESC-conditioned medium is a work in progress, we report here the ability to enrich these proteins using the heparin binding domains, and thus to provide a novel approach to study these clinically relevant molecules. Our ability to enrich the pro-regenerative activity of the hESC-secreted proteins is particularly important, because these embryonic factors improved the regenerative capacity of not only muscle, but also enhanced proliferation of neural progenitor cells, suggesting their possible ability to combat tissue degenerative disorders in multiple organ systems. Furthermore, hESC-secreted proteins exhibited not only proliferative effects on different progenitor cell types, but also neuroprotective effects on human cortical neurons in an *in vitro* model of Alzheimer's disease. These results suggests that hESC-produced molecules either prevent the death of human cortical neurons in an Aβ induced neurotoxic environment or are able to reduce the effect of Aβ toxicity by preventing the interaction of such toxic species with neuronal phenotypes that highly susceptible to Aβ, both possibilities that are clinically relevant outcomes and would be very interesting to study further.

With respect to the enhancement of myogenesis, this work revealed a pro-myogenic effect of mTeSR-1, which was linked to the high levels of FGF-2, a known inducer of proliferation in multiple cell types. Importantly, we show that the pro-myogenic activity of the hESC-secreted proteins manifests without added FGF-2 and that the hESC-conditioned Opti-MEM, which we typically use, does not contain any residual activity that is derived from mTeSR1.

While we found that the levels of FGF-2 protein are indeed elevated in old myofibers (in agreement with Chakkalakal et al.), signaling downstream of FGF signaling, namely pERK, was low and not different between the young and aged muscle stem cells or muscle fibers. In resolution of this interesting conundrum, we found that while in the old muscle this protein is localized intracellularly within the myofibers (away from its receptors and from satellite cells), in the young muscle, FGF-2 is located mostly extracellularly in the basement membrane, which is the niche of muscle stem cells. As such, this work suggests that much more FGF-2 ligand is available for signaling to young muscle stem cells than in old muscle, but it is still unclear why FGF-2 does not induce proliferation of quiescent satellite cells in uninjured *young* muscle. Potentially, the disruption of the basement membrane due to the injury or attrition of the myofibers, differentiation of satellite cells along myogenic lineage, and/or extracellular matrix - based activation of FGF-2 for binding to its receptors might be required for the induction of FGF-2 signaling in the muscle stem cells responding to tissue damage. In support of this conclusion, FGF-2 had a weak effect on the proliferation of quiescent muscle stem cells derived from non-injured mice (both, young and old); and thus, not FGF-2 alone but other factors and signaling pathways are likely required for the breakage of satellite cell quiescence [5]. In support of this model, the MM14 myoblast line was found to be responsive to exogenous FGF-2 ligand, but not endogenous, and ectopic expression of oncogenic *ras* was found to be required to liberate or activate the endogenous extracellular FGF-2 for signaling [28]. Low numbers (~3-4%) of proliferating Ki67+ young satellite cells are explained by the fact that these cells (expected to be 99.1% quiescent) were cultured overnight in their own young serum that is known to be pro-proliferative [29]; proliferation of the aged quiescent satellite cells derived from uninjured muscle and cultured with old serum was very low, which is consistent with the fact that old satellite cells divide very poorly in the presence of aged serum [14, 30-32].

FGF-2 does not have a signal peptide, and the mechanisms of FGF-2 activation are not well described in general or in skeletal muscle [26, 29]; therefore, further work is required to understand the age-dependent defect in the localization and activation of FGF-2 signal transduction in muscle stem and progenitor cells. Notably, differential localization of FGF-2 might introduce experimental artifacts into its detection, since the basement membranes of myofibers typically become digested during muscle dissociation, and the plasma membrane may be damaged [26, 29]; thus, the identification of the precise levels of FGF-2 in sub-cellular compartments of skeletal muscle is not an easy task.

Importantly, our data directly demonstrate that the numbers of proliferating (Ki67+Pax7+) muscle stem cells do not increase with age, which is further corroborated by the lack of age-specific increase of BrdU+ muscle stem cells after 4-6 weeks of *in vivo* delivery of BrdU to young and old mice (Amy Wagers, personal communications). Summarily, it is inconsistent with our data that FGF-2 promotes proliferation of quiescent muscle stem cells in old mice, though it still may be possible that old satellite cells are lost through an indirect and MAPK-independent activity of FGF-2.

As well established, in response to injury or attrition of myofibers, quiescent muscle stem cells activate to divide, form myogenic lineage, and regenerate the tissue; and this process becomes inefficient with age. The work presented here introduces novel strategies for the purification and clinical use of the proteins that are able to rejuvenate the aged niches of organ stem cells and uncovers that the viability of differentiated cells in pathological tissues might be also enhanced by these clinically-relevant molecules.

## METHODS

### Animals

Young (2-3 month old) and old (22-24 month old) C57BL6/J mice were purchased from the Jackson Laboratory and the NIH. The animal experimental procedures were performed in accordance with the Guide for Care and Use of Laboratory Animals of the National Institutes of Health, and approved by the Office of Laboratory Animal Care, UC Berkeley.

### Antibodies

Antibodies for phospho-ERK1/2, ERK1/2, and cleaved caspase 3 were purchased from Cell Signaling. Laminin and Actin antibodies were from Sigma. FGF2 antibody was from Santa Cruz, Pax7 and eMyHC antibodies were from Hybridoma Bank, BrdU was from Abcam, and Map2 antibody was from BD Biosciences.

### Muscle fibers and muscle stem cell isolation

Uninjured TA muscle was dissected from healthy young and old mice and incubated at 37C in digestion medium (250 U/mL Collagenase type II in DMEM medium, buffered with 30 mM HEPES) for 1 hour [26]. Digested muscle was gently triturated and myofibers were collected. Myofibers were further digested with 1 U/mL Dispase and 40 U/mL Collagenase type II to liberate muscle stem cells [29]. Muscle stem cells were cultured in DMEM with serum from the same age mouse.

### Immunofluorescence analysis

Cells were fixed with 4% PFA for 10 minutes before permeablization with 0.1% Triton-X 100 for 30 minutes. Then cells were then immunostained for Pax7 (Hybridoma Bank) and ki67 (Abcam). Primary antibodies used for staining cortical human neurons were: mouse anti-MAP2 (1:500, BD Biosciences), rabbit anti-cleaved caspase 3 (1:100, Cell Signaling). For muscle section immunostaining, an uninjured TA muscle was sectioned at 10 um and stained for FGF2 (Santa Cruz) and laminin (Sigma).

### Western blotting

Muscle stem cells or myofibers were lysed in RIPA buffer containing 1X protease inhibitor (Roche). The protein concentration was determined by Bradford assay (Bio-Rad). Cell or fiber lysates were resuspended in 1X Laemmli buffer (Rio-rad), boiled for 5 minutes and separated on precast TGX gels from Biorad. The proteins were then transferred to PVDF membrane (Millipore) and blotted with the desired antibodies.

### Cell Culture

Rat NPCs were cultured in DMF12 (Gibco) with 5% N2 and 10 ng/mL FGF2, on laminin and polyornithine coated plates. For experimental conditions, cells were plated at 40,000 cells/well in coated 8-well chamber slides and cultured for 12-16 hours at 37C in 10% CO2 incubator prior to fixation with 70% ethanol at 4C. Adult human myoblasts were cultured and expanded in human growth medium (Ham's F-10 (Gibco), 10% Bovine Growth Serum (Hyclone), 30 ng/mL FGF2, and 1% penicillin-streptomycin on Matrigel (BD Biosciences) coated plates (1:100 matrigel:PBS), at 37C and 5% CO2. For experimental conditions, cells were plated at 10,000 cells/well in Matrigel coated 8-well chamber slides (1:100 Matrigel: PBS), and cultured for 72 hours with daily re-feedings at 37C in 10% CO2 incubator prior to fixation with 70% ethanol at 4C. Mouse myoblasts were cultured and expanded in mouse growth medium: Ham's F-10 (Gibco), 20% Bovine Growth Serum (Hyclone), 5 ng/mL FGF2 and 1% penicillin-streptomycin on Matrigel coated plates (1:300 matrigel: PBS), at 37C and 5% CO2. For experimental conditions, cells were plated at 40,000 cells/well on Matrigel coated 8-well chamber slides (1:100 matrigel: PBS) and cultured for 24 hours at 37C in 10% CO2 incubator prior to fixation with 70% ethanol at 4C.

Human embryonic stem cells (H9 and H7 lines), were cultured on diluted Matrigel (1:30), in mTeSR-1 (Stem Cell Technologies), according to manufacturer's recommendations. hESCs were differentiated after plating in mTeSR-1 by changing the medium to DMEM/F12 with 10% Bovine Growth Serum (Hyclone), and culturing for an additional 6-8 days. Cells were washed with Opti-MEM (Gibco) and then cultured in Opti-MEM for 18 hours prior to collection as hESC-Conditioned Opti-MEM (hESC-Conditioned Medium) and stored at −80C.

All experiments using a MEK inhibitor were treated with 10 micromolar MEK1/2 Inhibitor (U0126, Cell Signaling Technologies).

### Cell culture and cortical differentiation of human pluripotent stem cells

The H1 (WiCell) and hESC line was cultured on Matrigel-coated cell culture plates (BD) in mTeSR-1 maintenance medium (Stem Cell Technologies). In adherent conditions, hPSCs were seeded at a density of 5×104 cells/cm2 in growth medium. At 50% confluence, the medium was gradually changed to neural basal medium (Invitrogen) containing N2 and B27 (Invitrogen). SMAD signaling inhibitors LDN193189 (Stemgent, 1 μM) and SB432542 (Tocris Biosciences, 10 μM) were added from day 1 to day 7 of neural induction. Cyclopamine (Calbiochem, 400 ng/ml) and FGF-2 (Peprotech, 10 ng/ml) were added from days 3-14 of differentiation. After 12-14 days, cells were mechanically passaged into poly-L-ornithine (Sigma Aldrich) and laminin (Invitrogen, 20μg/ml) coated plates and allowed to undergo maturation for 3-6 weeks. BDNF (10 ng/ml, Peprotech) was added to cultures one week after initiation of neuronal maturation. For EB mediated neural differentiation, PSCs were aggregated for 4 days in ultra low-attachment plates (Corning) and then seeded on Matrigel-coated plates. Cyclopamine (5 μM) and FGF-2 (10 ng/ml) were added to the cultures the following day until day 12 of neural induction. At day 14, structures with a rosette-like morphology were mechanically isolated and plated on poly-L-ornithine and laminin coated plates and allowed to undergo neuronal maturation for 4 weeks. BDNF (10 ng/ml) was added to the cultures one week after rosette isolation.

### Globulomer Preparation

The A-beta42 globulomer was prepared as described [33, 34]. Alkaline pretreatment of A-beta42 and preparation of low molecular weight A-beta by filtration protocols were used before beginning the globulomer preparation. After the 18-20 h incubation, the globulomer sample were concentrated to ~500 M via centrifugation and dialyzed into PBS before centrifuging the sample at 10,000 g for 10 min to remove aggregates in the pellet. The supernatant was saved, and the absorbance was measured at 276 nm wavelength to measure the concentration (extinction coefficient = 1390 M-1 cm-1).

### Immunocytochemistry

For immunoflourescence assays, mouse myoblasts were given a 2 hour 300μM BrdU pulse, respectively. Cells were then permeabilized in PBS + 0.25% Triton X-100 and incubated with primary antibodies overnight at 4C in PBS +2%FBS. Antigen retrieval was performed via a 10 minute 4 N HCl treatment followed by PBS washes. Primary staining was performed overnight with species-specific monoclonal antibodies for mouse anti-embryonic Myosin Heavy Chain (eMyHC, hybridoma clone 1.652, Developmental Studies Hybridoma Bank) and Rat-BrdU (Abcam Inc. ab6326), and desmin (Sigma-Aldrich DE-U-10 used at 1:300 for best discrimination of myogenic cells), for myoblasts and satellite progenitor cells, and Goat-Sox2 (Santa Cruz) for rNPCs. Secondary staining with fluorophore-conjugated, species-specific antibodies (Donkey anti-Rat-488, #712-485-150; Donkey anti-Mouse-488, #715-485-150; Donkey anti-Rat-Cy3 #712-165-150; or donkey anti-Mouse-Cy3 #715-165-150; all secondary antibodies from Jackson ImmunoResearch) was performed for 1 hour at room temperature at a 1:500 dilution in PBS +2%FBS. Nuclei were visualized by Hoechst staining, and samples were analyzed at room temperature with a Zeiss Axio Imager A1, and imaged with an Axiocam MRC camera and AxioVision software. Mouse myoblasts were imaged at 10X and 20X magnification, respectively. For cell quantification, 25-50 20x images per replicate were taken on the Molecular Devices ImageXpress Micro automated epifluorescence imager, followed by automated cell quantification using the multiwavelength cell scoring module within the MetaXpress analysis software.

### Heparin Binding of hESC-Secreted Proteins from hESC-Conditioned Medium

Heparin-Agarose Type I Beads (H 6508, Sigma Aldrich) were washed with molecular grade water and preconditioned in 1mL Opti-MEM as recommended by manufacturer. hESC-conditioned medium was incubated with Heparin-Agarose Beads for 2 hours shaking at 4C. Beads and all medium were separated by centrifugation. Myoblasts were treated with depleted medium after two rounds of centrifugation and separation of beads and medium so as to remove all residual beads from depleted hESC-conditioned medium.

After depleting hESC-Conditioned Opti-MEM, the protein bound heparin beads were washed two times for 10 minutes at 4C in 1ml PBS + .05% Tween-20. Proteins were eluted twice for 15 minutes at 4C in 400μl of elution buffer (.01M Tris-HCl pH 7.5 +1.5M NaCl + 0.1%BSA) to collect proteins in a total of 800μl of elution buffer. The proteins were purified by dialysis for 2 hours shaking at 4C in 500ml McCoy's 5A Medium (Gibco) followed by overnight dialysis shaking at 4C in 200ml Opti-MEM (Gibco). The eluted heparin beads were re-suspended in 800μl Opti-MEM and stored overnight at 4C. One hour after plating, mouse myoblasts were treated with respective mediums for 24 hours prior to 2 hour BrdU pulse and fixation in 70% ethanol.

### Muscle Injury

Isoflurane was used to anesthetize the animal during the muscle injury procedure. For bulk myofiber satellite cell activation, gastrocnemius muscles were injected with cardiotoxin 1 (Sigma) dissolved at 100 micrograms per milliliter in PBS, at 4 sites of 10 microliters each for each muscle. Muscles were harvested 3 days later. For focal injury, to assay regeneration *in vivo*, 5 microliters of 0.5 milligram per milliliter CTX was injected at two sites to the middle of the tibialis anterior, and muscle harvested 5 days later.

### Tissue Immunofluorescence and Histological Analysis

Muscle tissue was dissected, flash frozen in OCT compound (Tissue Tek; Sakura ) and cryo-sectioned at 10 micrometers, as previously described (Conboy et al., 2003). Cryo-sectioning was performed through the entire volume of muscle (typically 50-70 sections total, done at 200 μm intervals), thereby serially reconstituting the entire tissue, ex vivo. Muscle sections were stained with aqueous hematoxylin and eosin (H&E), as per the manufacturer's instructions (Sigma-Aldrich). Regeneration and myogenic potential was quantified by examining injury sites from representative sections along the muscle (spanning the volume of injury), then by measuring the injured/rege- nerating area using Adobe Photoshop Elements. Myofiber regeneration was quantified by counting total newly regenerated fibers and dividing by the regeneration area. Immunostaining was performed as described [35]. Briefly, after permeabilization in PBS + 1% FBS + 0.25% Triton-X-100, tissues and cells were incubated with primary antibodies in staining buffer (PBS + 1% FBS) for 1 h at room temperature, followed by 1 h incubation fluorochrome-labeled secondary antibodies (ALEXA at 1:1000). BrdU-specific immunostaining required an extra step of 2 M HCl treatment before permeablization.

### Quantification and Statistical Analysis

For quantification of immunofluorescent images, 25-100 20x images per replicate were taken on the Molecular Devices ImageXpress Micro automated epifluorescence imager, followed by automated cell quantification using the multiwavelength cell scoring module within the MetaXpress analysis software. Data was analyzed, using Anova and P values equal or lower than 0.05 were considered statistically significant.

## SUPPLEMENTARY FIGURES


