# Supplementary material for: hESC-secreted proteins can be enriched for multiple regenerative therapies by heparin-binding
Source: Aging (Albany NY). 2013 May 23;5(5):357–72. doi: 10.18632/aging.100559 (PMC3701111; doi:10.18632/aging.100559)
Supplement: Supplementary file 1 [file aging-05-357-s001.pdf]

SUPPLEMENTARY FIGURES

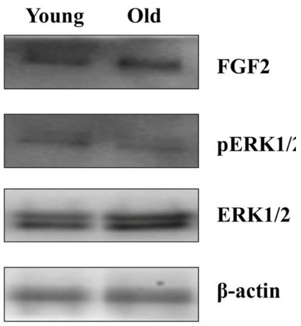

Supplementary Figure 1. Expression of FGF2 in quiescent muscle stem cells from young and old mice. Muscle stem cells were isolated from young and old uninjured muscle, as described in Methods. The cells were immediately lysed for Western blotting without culturing and the expression of FGF2, phosphor-ERK1/2, ERK1/2 and β-actin were analyzed. 30 minutes of enhanced chemiluminescence exposure was used for detection of FGF-2, while pERK, total ERK and actin were detected after 2min, 30 sec and 30 sec exposure, respectively. Low and age-independent levels of FGF-2 and pERK were detected in satellite cells that were derived from uninjured young and old TA muscle.

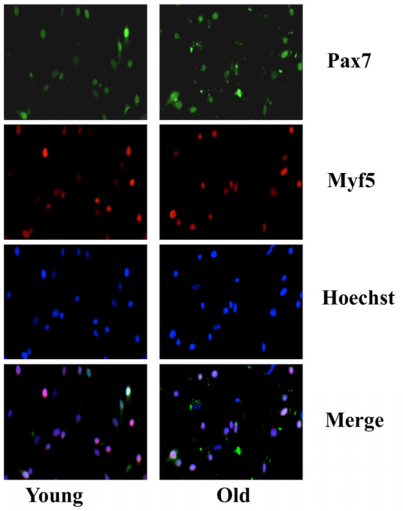

Supplementary Figure 2. Myogenic marker expression in young and old muscle stem cells. Muscle stem cell isolated from young and old mice were cultured for 24 hours and then immunostained for myogenic markers Pax7 and Myf-5. ~95% of isolated young and old satellite cells expressed these myogenic markers, demonstrating high and age-independent purity.

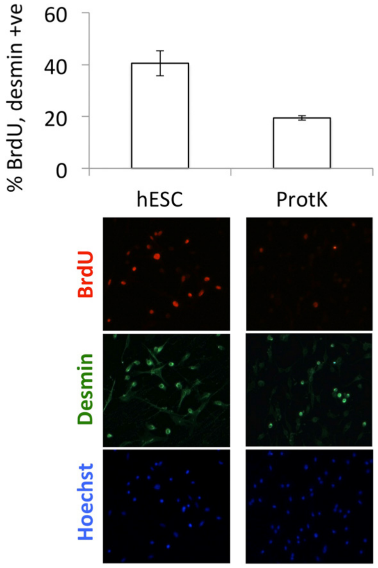

Supplementary Figure 3. Proteinase K treatement abolishes proliferative hESC factors. Old injury-activated satellite cells with associated myofibers were cultured overnight in 50% Opti-MEM with 10% old serum and 50% hESC conditioned Opti-MEM that was treated with pre-washed Proteinase K agarose beads (Sigma-Aldrich), for 1 hour at 37C followed by bead removal, or mock-treated hESC conditioned Opti-MEM. Cells received a 2 hour BrdU pulse to label proliferating cells before cell fixation. Immuno-fluorescence was performed for Desmin (green) and BrdU (red), with Hoechst (blue) labeling all cell nuclei. Proliferating, desmin+ve cells were quantified by imaging and scoring multiple random microscopic fields of each condition. Results are displayed as the mean percent of BrdU+,Desmin+ proliferating satellite cell cells +/- SEM, p<0.005, n=3 replicate experiments.
